# Hazards of administration of benzodiazepines in patients with adaptive hyperventilation: a case rapport

**DOI:** 10.1192/j.eurpsy.2022.1504

**Published:** 2022-09-01

**Authors:** S. Petrykiv, M. Arts, L. De Jonge

**Affiliations:** 1 GGZWNB, Psychiatry, Halsteren, Netherlands; 2 GGZWNB, Psychiatry, Bergen op Zoom, Netherlands; 3 Leonardo Scientific Research Institute, Neuropsychiatry, Bergen op Zoom, Netherlands

**Keywords:** Benzodiazepines, Hypoventilation, Case rapport, Treatment

## Abstract

**Introduction:**

Only three population-based observational human studies provided evidence that benzodiazepines (BZD) are associated with clinically adverse respiratory outcome. Striking was the finding that BZD drug exposure was associated with a 32% significantly increased adjusted risk of all-cause mortality, including, of note, the subgroup of individuals with no comorbidities. Causation, however, cannot be inferred in observational study design and, highly likely, recipients received BZD’s in these studies to help treating anxiety related to inter alia pre-existing respiratory symptoms.

**Objectives:**

Based on one fatal particular case, authors of current rapport explain what can go wrong when BZD’s are given in patient with respiratory impairment.

**Methods:**

Authors provide a model on how an increase in carbon dioxide can lead to impaired cerebral autoregulation in a person with pre-existing respiratory decompensation. Discussion of integrative metabolic and vascular physiology.

**Results:**

Case rapport of a 18 y.o. otherwise healthy man, who was hospitalized with a novel episode of diabetic ketoacidosis accompanied by profound hypocapnia and anxiety, and who deteriorated and died shortly after airway management because of a clinically important acid-base balance disturbance caused by increased carbon dioxide. All the blood tests and results of respiratory monitoring were collected and carefully assessed.

**Conclusions:**

Current case suggests that the P(CO(2))--HCO(3) hypothesis is consistent with known data on impaired cerebral autoregulation in diabetic ketoacidosis, driven mainly by increased levels of pCO2. In our opinion, it indicates the recommendation not to administrate BZD’s in patients with pre-existing compensatory hyperventilation as it may counter to the logic of adaptive physiology.

**Disclosure:**

No significant relationships.

